# Supplementary material for: Sporting tournaments and changed birth rates 9 months later: a systematic review
Source: PeerJ. 2024 Feb 27;12:e16993. doi: 10.7717/peerj.16993 (PMC10906258; doi:10.7717/peerj.16993)
Supplement: Supplemental Information 4 [file peerj-12-16993-s004.docx]

1. Rationale for Conducting the Systematic Review / Meta-analysis:

This systematic review responds to a critical gap in the literature by investigating the relationship between major sporting tournaments and subsequent changes in birth patterns. While prior research has highlighted the association between celebratory events and increased birth rates, the impact of major sporting tournaments on birth metrics remained insufficiently explored. The distinct emotional intensification during these tournaments, potentially influencing hormonal shifts and sexual behaviour, warranted a comprehensive investigation to establish the extent of their influence on birth rates and sex ratios. By synthesizing existing evidence, we aimed to provide a more nuanced understanding of the implications of these societal events for population demographics and healthcare planning.

2. Contribution to Knowledge in Light of Previously Published Reports:

Our systematic review contributes to knowledge by consolidating and analysing data from various studies worldwide to demonstrate that major sporting tournaments significantly impact birth patterns. While previous reports have touched on increased births after celebratory events, this study uniquely focuses on the context of sporting tournaments, revealing consistent associations across different sports and continents. By systematically examining various tournaments, including the Super Bowl, UEFA Championships, FIFA World Cup, and Rugby World Cup, we establish a clear link between these events and altered birth metrics. This review expands upon existing literature and underscores the need to consider broader societal events in understanding demographic trends, offering implications for healthcare resource allocation and further emphasising the role of emotions in shaping population dynamics.
